# Fourier series of higher-order Daehee and Changhee functions and their applications

**DOI:** 10.1186/s13660-017-1427-7

**Published:** 2017-06-24

**Authors:** Dongkyu Lim

**Affiliations:** School of Mathematical Sciences, Nankai University, Tianjin City, 300071 China

**Keywords:** 11B68, 42A16, Fourier series, Daehee polynomials, Changhee polynomials, Bernoulli functions, Daehee functions, Changhee functions

## Abstract

In the paper, the author considers the Fourier series related to higher-order Daehee and Changhee functions and establishes some new identities for higher-order Daehee and Changhee functions.

## Introduction and main results

It is common knowledge that the Bernoulli polynomials $B_{n}(x)$ and the Euler polynomials $E_{n}(x)$ for $n\geq0$ can be generated by

$$ \begin{aligned} \frac{t}{e^{t}-1}e^{xt}&=\sum _{n=0}^{\infty}B_{n}(x)\frac{t^{n}}{n!} \end{aligned} $$

and

$$ \begin{aligned} \frac{2}{e^{t}+1}e^{xt}=\sum _{n=0}^{\infty}E_{n}(x)\frac{t^{n}}{n!}, \end{aligned} $$

respectively (see [1–23]).

With the viewpoint of deformed Bernoulli polynomials, the Daehee polynomials $D_{n}(x)$ for $n\geq0$ are defined by the generating function to be

1 $$ \frac{\log{(1+t)}}{t}(1+t)^{x}=\sum _{n=0}^{\infty}D_{n}(x)\frac{t^{n}}{n!}.$$

It is easy to see that the generating function of the Daehee polynomials $D_{n}(x)$ can be reformed as

$$ \frac{\log{(1+t)}}{t}(1+t)^{x}=\frac{\log{(1+t)}}{e^{\log {(1+t)}}-1}e^{x\log{(1+t)}}. $$

From (1), we note that

2 $$ \begin{aligned}[b] \frac{\log{(1+t)}}{e^{\log{(1+t)}}-1}e^{x\log{(1+t)}}&=\sum _{n=0}^{\infty}B_{n}(x) \frac{1}{n!}\bigl(\log{(1+t)}\bigr)^{n} \\ &=\sum_{n=0}^{\infty}B_{n}(x)\sum _{m=n}^{\infty}S_{1}(m,n) \frac {t^{m}}{m!} \\ &=\sum_{m=0}^{\infty} \Biggl(\sum _{n=0}^{m}B_{n}(x)S_{1}(m,n) \Biggr)\frac{t^{m}}{m!}, \end{aligned} $$

where $S_{1}(m,n)$ stands for the Stirling number of the first kind which is defined as

$$ (x)_{0}=1, \qquad(x)_{n}=x(x-1)\cdots(x-n+1)=\sum _{l=0}^{n}S_{1}(n,l)x^{l}\quad (n \geq1). $$

Combining (1) with (2) yields the following relation:

$$ D_{m}(x)=\sum_{n=0}^{m}B_{n}(x)S_{1}(m,n)\quad (m\geq0). $$

By replacing *t* by $e^{t}-1$ in (1), we can derive

3 $$ \begin{aligned}[b] \sum_{n=0}^{\infty}B_{n}(x) \frac{t^{n}}{n!}&=\frac{t}{e^{t}-1}e^{xt}=\sum _{m=0}^{\infty}D_{m}(x)\frac{1}{m!} \bigl(e^{t}-1\bigr)^{m} \\ &=\sum_{m=0}^{\infty}D_{m}(x)\sum _{n=m}^{\infty}S_{2}(n,m) \frac {t^{n}}{n!} \\ &=\sum_{n=0}^{\infty} \Biggl(\sum _{m=0}^{n}D_{n}(x)S_{2}(n,m) \Biggr)\frac{t^{n}}{n!}, \end{aligned} $$

where $S_{2}(n,m)$ is the Stirling number of the second kind which is given by $x^{n}= \sum_{l=0}^{\infty}S_{2}(n,l)(x)_{l}$ $(n\geq0)$.

Comparing the coefficients on the both sides of (3), we obtain

$$ B_{n}(x)=\sum_{m=0}^{n}D_{m}(x)S_{2}(n,m)\quad (n\geq0). $$

Also, with the viewpoint of deformed Euler polynomials, the Changhee polynomials $\mathit{Ch}_{n}(x)$ for $n\geq0$ are defined by the generating function to be

4 $$ \frac{2}{2+t}(1+t)^{x}=\sum _{n=0}^{\infty}\mathit{Ch}_{n}(x)\frac{t^{n}}{n!}.$$

Definition (4) can be written as

$$ \begin{aligned} \frac{2}{e^{\log{(1+t)}}+1}e^{x\log{(1+t)}}&=\sum _{n=0}^{\infty }E_{n}(x)\frac{1}{n!}\bigl( \log{(1+t)}\bigr)^{n} \\ &=\sum_{n=0}^{\infty}E_{n}(x)\sum _{m=n}^{\infty}S_{1}(m,n) \frac{t^{m}}{m!} \\ &=\sum_{m=0}^{\infty} \Biggl(\sum _{n=0}^{m}E_{n}(x)S_{1}(m,n) \Biggr)\frac{t^{m}}{m!}. \end{aligned} $$

Combination of this identity with (4) results in the following relation:

$$ \mathit{Ch}_{m}(x)=\sum_{n=0}^{m}E_{n}(x)S_{1}(m,n)\quad (m\geq0). $$

Now replacing *t* by $e^{t}-1$ in (4), we have

$$ \begin{aligned} \sum_{n=0}^{\infty}E_{n}(x) \frac{t^{n}}{n!}&=\frac{2}{e^{t}+1}e^{xt}=\sum _{m=0}^{\infty}\mathit{Ch}_{m}(x)\frac{1}{m!} \bigl(e^{t}-1\bigr)^{m} \\ &=\sum_{m=0}^{\infty}\mathit{Ch}_{m}(x)\sum _{n=m}^{\infty}S_{2}(n,m) \frac {t^{n}}{n!} \\ &=\sum_{n=0}^{\infty} \Biggl(\sum _{m=0}^{n}\mathit{Ch}_{n}(x)S_{2}(n,m) \Biggr)\frac{t^{n}}{n!}. \end{aligned} $$

Equating coefficients on the very ends of the above identity leads to

$$ E_{n}(x)=\sum_{m=0}^{n}\mathit{Ch}_{m}(x)S_{2}(n,m)\quad (n\geq0). $$

In recent decades, many mathematicians have investigated some interesting extensions or modifications of the Daehee and Changhee polynomials along with related combinatorial identities and their applications (see [4, 9, 10, 14, 16, 17, 19, 23]). Especially, Kim and his coauthors have studied the Fourier series related to various types of Bernoulli functions in [7, 11–13, 15]. The purpose of this paper is to study the Fourier series related to higher-order Daehee and Changhee functions and establish some new identities for higher-order Daehee and Changhee functions.

For any real number *x*, we define

$$ \langle x\rangle=x-[x]\in(0,1), $$

where $[x]$ is the integer part of *x*. Then $D_{n}(\langle x\rangle)$ are functions defined on $(-\infty,\infty)$ and periodic with period 1, which are called Daehee functions.

For $r\in\mathbb{N}$ and $n\geq0$, we note that the higher-order Daehee polynomials $D^{(r)}_{n}(x)$ and the higher-order Changhee polynomials $\mathit{Ch}^{(r)}_{n}(x)$ may also be represented by the following generating function:

5 $$ \biggl(\frac{\log{(1+t)}}{t} \biggr)^{r}(1+t)^{x}= \sum_{n=0}^{\infty }D^{(r)}_{n}(x) \frac{t^{n}}{n!} $$

and

6 $$ \biggl(\frac{2}{2+t} \biggr)^{r}(1+t)^{x}= \sum_{n=0}^{\infty }\mathit{Ch}^{(r)}_{n}(x) \frac{t^{n}}{n!}, $$

respectively (see [4, 10, 14]). When $x=0$, $D^{(r)}_{n}=D^{(r)}_{n}(0)$ are called the higher-order Daehee numbers and $\mathit{Ch}^{(r)}_{n}=\mathit{Ch}^{(r)}_{n}(0)$ are called the higher-order Changhee numbers. And it is easy to see that

$$ D^{(1)}_{n}(x)=D_{n}(x),\qquad \mathit{Ch}^{(1)}_{n}(x)=\mathit{Ch}_{n}(x). $$

Then $D^{(r)}_{n}(\langle x\rangle)$ and $\mathit{Ch}^{(r)}_{n}(\langle x\rangle)$ are functions defined on $(-\infty,\infty)$ and periodic of period 1, which are called Daehee functions of order *r* and Changhee functions of order *r*, respectively.

Recall from [15, 24] that the Bernoulli function may be represented by

7 $$ \begin{aligned} B_{m}\bigl(\langle x\rangle \bigr)=-m!\sum_{\substack{n=-\infty\\ n\neq0}}^{\infty }\frac{e^{2\pi inx}}{(2\pi in)^{m}} \quad(m\geq2) \end{aligned} $$

and

8 $$ \begin{aligned} -m!\sum_{\substack{n=-\infty\\ n\neq0}}^{\infty} \frac{e^{2\pi inx}}{(2\pi in)^{m}}= \textstyle\begin{cases} B_{1}(\langle x\rangle)& \text{for $x\notin\mathbb{Z}$},\\ 0 & \text{for $x\in\mathbb{Z}$}. \end{cases}\displaystyle \end{aligned} $$

The Fourier series expansion of the Bernoulli functions is useful in computing the special values of the Dirichlet *L*-functions. For details, one is referred to [24].

Our main results in this paper can be stated as the following theorems.

### Theorem 1

*Let* $m\geq2$, $r\geq1$. *Assume that* $D^{(r)}_{m-1}=0$.

- $D^{(r)}_{m}(\langle x\rangle)$ *has the Fourier series expansion*
  $$ \begin{aligned} D^{(r)}_{m}\bigl(\langle x\rangle \bigr)=D^{(r)}_{m}-\sum_{\substack{n=-\infty\\ n\neq0}}^{\infty} \Biggl(\sum_{k=1}^{m}\frac{(m)_{k}}{(2\pi in)^{k}}D^{(r)}_{m-k} \Biggr){e^{2\pi inx}} \end{aligned} $$
  *for* $x\in(-\infty,\infty)$. *Here the convergence is uniform*.
- $D^{(r)}_{m}(\langle x\rangle)= \sum_{\substack{k=0\\ k\neq1}}^{m}\binom{m}{k}D^{(r)}_{m-1}B_{k}(\langle x\rangle)$, *for all* $x\in(-\infty,\infty)$, *where* $B_{k}(\langle x\rangle )$ *is the Bernoulli function*.

### Theorem 2

*Let* $m\geq2$, $r\geq1$. *Assume that* $D^{(r)}_{m-1}\neq0$.

- $$ \begin{aligned} D^{(r)}_{m}-\sum _{\substack{n=-\infty\\ n\neq0}}^{\infty} \Biggl(\sum_{k=1}^{m} \frac{(m)_{k}}{(2\pi in)^{k}}D^{(r)}_{m-k} \Biggr){e^{2\pi inx}}= \textstyle\begin{cases} D^{(r)}_{m}(\langle x\rangle)& \textit{for } x\notin\mathbb{Z},\\ D^{(r)}_{m}+\frac{m}{2}D^{(r)}_{m-1} & \textit{for } x\in\mathbb{Z}. \end{cases}\displaystyle \end{aligned} $$
  *Here the convergence is pointwise*.
- $$ \begin{aligned} \sum_{k=0}^{m} \binom{m}{k}D^{(r)}_{m-k}B_{k}\bigl(\langle x \rangle \bigr)=D^{(r)}_{m}(x)\quad\textit{for } x\notin \mathbb{Z} \end{aligned} $$
  *and*
  $$ \begin{aligned} \sum_{\substack{k=0\\ k\neq1}}^{m} \binom{m}{k}D^{(r)}_{m-k}B_{k}\bigl(\langle x \rangle\bigr)=D^{(r)}_{m}+\frac{m}{2}D^{(r)}_{m-1} \quad\textit{for } x\in \mathbb{Z}, \end{aligned} $$
  *where* $B_{k}(\langle x\rangle)$ *is the Bernoulli function*.

### Theorem 3

*Let* $m\geq2$, $r\geq1$. *Assume that* $\mathit{Ch}^{(r)}_{m}=\mathit{Ch}^{(r-1)}_{m}$.

- $\mathit{Ch}^{(r)}_{m}(\langle x\rangle)$ *has the Fourier series expansion*
  $$ \begin{aligned} \mathit{Ch}^{(r)}_{m}\bigl(\langle x \rangle\bigr)={}&\frac{2}{m+1} \bigl(\mathit{Ch}_{m+1}^{(r-1)}-\mathit{Ch}_{m+1}^{(r)} \bigr) \\ & +\sum_{\substack{n=-\infty\\ n\neq0}}^{\infty} \Biggl(\sum _{k=1}^{m}\frac{2(m)_{k-1}}{(2\pi in)^{k}} \bigl(\mathit{Ch}_{m-k+1}^{(r)}-\mathit{Ch}_{m-k+1}^{(r-1)} \bigr) \Biggr){e^{2\pi inx}} \end{aligned} $$
  *for* $x\in(-\infty,\infty)$. *Here the convergence is uniform*.
- $$ \begin{aligned} \mathit{Ch}^{(r)}_{m}\bigl(\langle x \rangle\bigr)={}&\frac{2}{m+1} \bigl(\mathit{Ch}_{m+1}^{(r)}-\mathit{Ch}_{m+1}^{(r)} \bigr) \\ & +\sum_{k=1}^{m}\frac{2(m)_{k-1}}{k!} \bigl(\mathit{Ch}_{m-k+1}^{(r-1)}-\mathit{Ch}_{m+1}^{(r)} \bigr)B_{k}\bigl(\langle x\rangle\bigr)\quad \textit{for } x\notin \mathbb{Z} \end{aligned} $$
  *and*
  $$ \begin{aligned} \mathit{Ch}^{(r)}_{m}\bigl(\langle x \rangle\bigr)={}&\frac{2}{m+1} \bigl(\mathit{Ch}_{m+1}^{(r-1)}-\mathit{Ch}_{m-k+1}^{(r)} \bigr) \\ & +\sum_{k=2}^{m}\frac{2(m)_{k-1}}{k!} \bigl(\mathit{Ch}_{m-k+1}^{(r-1)}-\mathit{Ch}_{m+1}^{(r)} \bigr)B_{k}\bigl(\langle x\rangle\bigr)\quad \textit{for } x\in \mathbb{Z}, \end{aligned} $$
  *where* $B_{k}(\langle x\rangle)$ *is the Bernoulli function*.

### Theorem 4

*Let* $m\geq1$, $r\geq1$. *Assume that* $\mathit{Ch}^{(r)}_{m}\neq \mathit{Ch}^{(r-1)}_{m}$.

- $$ \begin{gathered} \frac{2}{m+1} \bigl(\mathit{Ch}^{(r-1)}_{m+1}-\mathit{Ch}^{(r)}_{m+1} \bigr)+\sum_{\substack{n=-\infty\\ n\neq0}}^{\infty} \Biggl(\sum _{k=1}^{n}\frac {(m)_{k-1}}{(2\pi in)^{k}} \bigl(\mathit{Ch}^{(r)}_{m-k+1}-\mathit{Ch}^{(r-1)}_{m-k+1} \bigr) \Biggr){e^{2\pi inx}} \\ \quad = \textstyle\begin{cases} \mathit{Ch}^{(r)}_{m}(\langle x\rangle)& \textit{for } x\notin\mathbb{Z},\\ \mathit{Ch}^{(r-1)}_{m} & \textit{for } x\in\mathbb{Z}. \end{cases}\displaystyle \end{gathered} $$
  *Here the convergence is pointwise*.
- $$ \begin{gathered} \frac{2}{m+1} \bigl(\mathit{Ch}_{m+1}^{(r-1)}-\mathit{Ch}_{m+1}^{(r)} \bigr) +\sum_{k=1}^{m}\frac{2(m)_{k-1}}{k!} \bigl(\mathit{Ch}_{m-k+1}^{(r-1)}-\mathit{Ch}_{m-k+1}^{(r)} \bigr)B_{k}\bigl(\langle x\rangle \bigr)\\ \quad=\mathit{Ch}^{(r)}_{m} \bigl(\langle x\rangle\bigr)\quad\textit{for } x\notin\mathbb{Z} \end{gathered} $$
  *and*
  $$ \begin{gathered} \frac{2}{m+1} \bigl(\mathit{Ch}_{m+1}^{(r-1)}-\mathit{Ch}_{m+1}^{(r)} \bigr) +\sum_{k=2}^{m}\frac{2(m)_{k-1}}{k!} \bigl(\mathit{Ch}_{m-k+1}^{(r-1)}-\mathit{Ch}_{m-k+1}^{(r)} \bigr)B_{k}\bigl(\langle x\rangle \bigr)\\ \quad=\mathit{Ch}^{(r-1)}_{m} \bigl(\langle x\rangle\bigr)\quad\textit{for } x\in\mathbb{Z}, \end{gathered} $$
  *where* $B_{k}(\langle x\rangle)$ *is the Bernoulli function*.

## Proofs of Theorems 1-4

We are now in a position to prove our four theorems.

By analyzing definition (5), we have

$$ D^{(r)}_{m}(x+1)=D^{(r)}_{m}(x)+mD^{(r)}_{m-1}(x) \quad(m\geq0). $$

Furthermore, we observe that

$$ \begin{aligned} \sum_{m=0}^{\infty}D^{(r)}_{m}(x) \frac{t^{m}}{m!}&= \biggl(\frac{\log {(1+t)}}{t} \biggr)^{r}(1+t)^{x+1} \\ &= \biggl(\frac{\log{(1+t)}}{t} \biggr)^{r}(1+t)^{x}+ \biggl( \frac{\log {(1+t)}}{t} \biggr)^{r}(1+t)^{x}t \\ &=\sum_{m=0}^{\infty}D^{(r)}_{m}(x) \frac{t^{m}}{m!}+\sum_{m=0}^{\infty }D^{(r)}_{m}(x) \frac{t^{m+1}}{m!} \\ &=\sum_{m=0}^{\infty}\bigl(D^{(r)}_{m}(x)+mD^{(r)}_{m-1}(x) \bigr)\frac{t^{m}}{m!}. \end{aligned} $$

Letting $x=0$ in the above equation leads to

$$ D^{(r)}_{m}(1)=D^{(r)}_{m}+mD^{(r)}_{m-1} \quad(m\geq0). $$

Now, we assume that $m,r\geq1$. $D^{(r)}_{m}(\langle x\rangle)$ is piecewise $C^{\infty}$. Further, in view of (2), $D^{(r)}_{m}(\langle x\rangle)$ is continuous for those $(r,m)$ with $D^{(r)}_{m-1}=0$, and is discontinuous with jump discontinuities at integers for those $(r,m)$ with $D^{(r)}_{m-1}\neq0$. The Fourier series of $D^{(r)}_{m}(\langle x\rangle)$ may be represented by

$$ \sum_{n=-\infty}^{\infty}C^{(r,m)}_{n}e^{2\pi inx} \quad(i=\sqrt{-1}), $$

where

9 $$ \begin{aligned}[b] C^{(r,m)}_{n}&= \int_{0}^{1}D^{(r)}_{m}\bigl( \langle x\rangle\bigr)e^{-2\pi inx}\,dx= \int_{0}^{1}D^{(r)}_{m}(x)e^{-2\pi inx}\,dx \\ &= \biggl[\frac{1}{m+1}D^{(r)}_{m+1}(x)e^{-2\pi inx} \biggr]^{1}_{0}+\frac {2\pi in}{m+1} \int_{0}^{1}D^{(r)}_{m+1}(x)e^{-2\pi inx}\,dx \\ &=\frac{1}{m+1} \bigl(D^{(r)}_{m+1}(1)-D^{(r)}_{m+1} \bigr)+\frac{2\pi in}{m+1}C^{(r,m+1)}_{n} \\ &=D^{(r)}_{m}+\frac{2\pi in}{m+1}C^{(r,m+1)}_{n}. \end{aligned} $$

Replacing *m* by $m-1$ in (9), we arrive at the following result:

$$ \begin{aligned} C^{(r,m-1)}_{n}=D^{(r)}_{m-1}+ \frac{2\pi in}{m}C^{(r,m)}_{n}. \end{aligned} $$

### Case 1

Let $n\neq0$. Then we acquire that

10 $$ \begin{aligned}[b] C^{(r,m)}_{n}={}& \frac{m}{2\pi in}C^{(r,m-1)}_{n}-\frac{m}{2\pi in}D^{(r)}_{m-1} \\ ={}&\frac{m}{2\pi in} \biggl(\frac{m-1}{2\pi in}C^{(r,m-2)}_{n}- \frac {m-1}{2\pi in}D^{(r)}_{m-2} \biggr)-\frac{m}{2\pi in}D^{(r)}_{m-1} \\ ={}&\frac{m(m-1)}{(2\pi in)^{2}}C^{(r,m-2)}_{n}-\frac{m(m-1)}{(2\pi in)^{2}}D^{(r)}_{m-2}- \frac{m}{2\pi in}D^{(r)}_{m-1} \\ ={}&\frac{m(m-1)}{(2\pi in)^{2}} \biggl(\frac{m-2}{2\pi in}C^{(r,m-3)}_{n}- \frac{m-2}{2\pi in}D^{(r)}_{m-3} \biggr) \\ & -\frac {m(m-1)}{(2\pi in)^{2}}D^{(r)}_{m-2}-\frac{m}{2\pi in}D^{(r)}_{m-1} \\ ={}&\frac{m(m-1)(m-2)}{(2\pi in)^{2}}C^{(r,m-3)}_{n}-\frac {m(m-1)(m-2)}{(2\pi in)^{3}}D^{(r)}_{m-3} \\ & -\frac{m(m-1)}{(2\pi in)^{2}}D^{(r)}_{m-2}-\frac{m}{2\pi in}D^{(r)}_{m-1} \\ ={}&\cdots \\ ={}&\frac{m(m-1)(m-2)\cdots2}{(2\pi in)^{m-1}}C^{(r,1)}_{n}-\sum _{k=1}^{m-1}\frac{(m)_{k}}{(2\pi in)^{k}}D^{(r)}_{m-k}. \end{aligned} $$

Moreover, we observe that

11 $$ \begin{aligned}[b] C^{(r,1)}_{n}&= \int_{0}^{1}D^{(r)}_{1}(x)e^{-2\pi inx}\,dx= \int _{0}^{1}\bigl(x+D^{(r)}_{1} \bigr)e^{-2\pi inx}\,dx \\ &= \int_{0}^{1}xe^{-2\pi inx}\,dx+D^{(r)}_{1} \int_{0}^{1}e^{-2\pi inx}\,dx \\ &=-\frac{1}{2\pi in} \bigl[xe^{-2\pi inx} \bigr]^{1}_{0}+ \frac{1}{2\pi in} \int_{0}^{1}e^{-2\pi inx}\,dx =-\frac{1}{2\pi in}. \end{aligned} $$

Combining (11) with (10), we immediately derive the following equation:

$$ C^{(r,m)}_{n}= \frac{m!}{(2\pi in)^{m}}-\sum_{k=1}^{m-1} \frac {(m)_{k}}{(2\pi in)^{k}}D^{(r)}_{m-k} =-\sum_{k=1}^{m}\frac{(m)_{k}}{(2\pi in)^{k}}D^{(r)}_{m-k}. $$

### Case 2

Let $n=0$. Then we have

$$ \begin{aligned} C^{(r,m)}_{0}&= \int_{0}^{1}D^{(r)}_{m}\bigl( \langle x\rangle\bigr)\,dx= \int _{0}^{1}D^{(r)}_{m}(x)\,dx \\ &=\frac{1}{m+1} \bigl[D^{(r)}_{m+1}(x) \bigr]^{1}_{0} \\ &=\frac{1}{m+1} \bigl(D^{(r)}_{m+1}(1)-D^{(r)}_{m+1} \bigr) =D^{(r)}_{m}. \end{aligned} $$

While that in (8) converges pointwise, the series in (7) converges uniformly. We assume that $D^{(r)}_{m-1}=0$. Then we have $D^{(r)}_{m}(1)=D^{(r)}_{m}$ for $m\geq2$. As $D^{(r)}_{m}(\langle x\rangle)$ is piecewise $C^{\infty }$ and continuous, the Fourier series of $D^{(r)}_{m}(\langle x\rangle )$ converges uniformly to $D^{(r)}_{m}(\langle x\rangle)$ and

12 $$ \begin{aligned}[b] D^{(r)}_{m}\bigl( \langle x\rangle\bigr)={}&\sum_{n=-\infty}^{\infty }C^{(r,m)}_{n}e^{2\pi inx} \\ ={}&D^{(r)}_{m}-\sum_{\substack{n=-\infty\\ n\neq0}}^{\infty} \Biggl(\sum_{k=1}^{m}\frac{(m)_{k}}{(2\pi in)^{k}}D^{(r)}_{m-k} \Biggr){e^{2\pi inx}} \\ ={}&D^{(r)}_{m}+\sum_{k=1}^{m} \frac{(m)_{k}}{k!}D^{(r)}_{m-k} \Biggl(k!\sum _{\substack{n=-\infty\\ n\neq0}}^{\infty}\frac{e^{2\pi inx}}{(2\pi in)^{k}} \Biggr) \\ ={}&D^{(r)}_{m}+\sum_{k=2}^{m} \binom{m}{k}D^{(r)}_{m-k}B_{k}\bigl(\langle x \rangle\bigr) +\binom{m}{1}D^{(r)}_{m-1}\times \textstyle\begin{cases} B_{1}(\langle x\rangle)& \text{for $x\notin\mathbb{Z}$},\\ 0 & \text{for $x\in\mathbb{Z}$} \end{cases}\displaystyle \\ ={}& \textstyle\begin{cases} \sum_{k=0}^{m}\binom{m}{k}D^{(r)}_{m-1}B_{k}(\langle x\rangle)& \text{for $x\notin\mathbb{Z}$},\\ \sum_{\substack{k=0\\ k\neq1}}^{m}\binom{m}{k}D^{(r)}_{m-1}B_{k}(\langle x\rangle) & \text{for $x\in\mathbb{Z}$}. \end{cases}\displaystyle \end{aligned} $$

Note that (12) holds whether $D^{(r)}_{m-1}=0$ or not. However, if $D^{(r-1)}_{m-1}=0$, then

$$ \begin{aligned} D^{(r)}_{m}\bigl( \langle x\rangle\bigr)= \sum_{\substack{k=0\\ k\neq1}}^{m} \binom {m}{k}D^{(r)}_{m-1}B_{k}\bigl(\langle x \rangle\bigr)\quad\text{for all $x\in (-\infty,\infty)$}. \end{aligned} $$

Therefore, we obtain the result in Theorem 1.

Assume next that $D^{(r)}_{m-1}\neq0$. Then we have $D^{(r)}_{m}(1)\neq D^{(r)}_{m}$ and hence $D^{(r)}_{m}(\langle x\rangle)$ is piecewise $C^{\infty}$ and discontinuous with jump discontinuities at integers. Thus the Fourier series of $D^{(r)}_{m}(\langle x\rangle)$ converges pointwise to $D^{(r)}_{m}(\langle x\rangle)$ for $x\notin\mathbb{Z}$, and converges to $\frac{1}{2} (D^{(r)}_{m}+D^{(r)}_{m}(1))=D^{(r)}_{m}+(m/2)D^{(r)}_{m-1}$ for $x\in \mathbb{Z}$. Finally, we obtain the formulas in Theorem 2.

From now on we focus on definition (6). Then we can find

13 $$ \mathit{Ch}^{(r)}_{m}(x+1)+\mathit{Ch}^{(r)}_{m}(x)=2\mathit{Ch}^{(r-1)}_{m}(x). $$

In other words,

$$ \begin{aligned} \sum_{m=0}^{\infty}\mathit{Ch}^{(r)}_{m}(x+1) \frac{t^{m}}{m!}&= \biggl(\frac {2}{2+t} \biggr)^{r}(1+t)^{x+1} \\ &=2 \biggl(\frac{2}{2+t} \biggr)^{r-1}(1+t)^{x}- \biggl(\frac{2}{2+t} \biggr)^{r}(1+t)^{x} \\ &=2\sum_{m=0}^{\infty}\mathit{Ch}^{(r-1)}_{m}(x) \frac{t^{m}}{m!}-\sum_{m=0}^{\infty }\mathit{Ch}^{(r)}_{m}(x) \frac{t^{m}}{m!} \\ &=\sum_{m=0}^{\infty} \bigl[2\mathit{Ch}^{(r-1)}_{m}(x)-\mathit{Ch}^{(r)}_{m}(x) \bigr]\frac{t^{m}}{m!}. \end{aligned} $$

Taking $x=0$ in (13) yields

$$ \begin{aligned} \mathit{Ch}^{(r)}_{m}(1)+\mathit{Ch}^{(r)}_{m}=2\mathit{Ch}^{(r-1)}_{m} \quad(m\geq0). \end{aligned} $$

This equation means that

$$ \begin{aligned} \mathit{Ch}^{(r)}_{m}=\mathit{Ch}^{(r)}_{m}(1)\quad \Leftrightarrow \quad\mathit{Ch}^{(r)}_{m}=\mathit{Ch}^{(r-1)}_{m}. \end{aligned} $$

Assume that $m\geq1$ and $r\geq1$ $\mathit{Ch}^{(r)}_{m}(\langle x\rangle)$ is piecewise $C^{\infty}$. In addition, $\mathit{Ch}^{(r)}_{m}(\langle x\rangle)$ is continuous for those $(r,m)$ with $\mathit{Ch}^{(r)}_{m}=\mathit{Ch}^{(r-1)}_{m}$ and discontinuous with jump discontinuities at integers for those $(r,m)$ with $\mathit{Ch}^{(r)}_{m}\neq \mathit{Ch}^{(r-1)}_{m}$. The Fourier series of $\mathit{Ch}^{(r)}_{m}(\langle x\rangle)$ is

$$ \begin{aligned} \sum_{n=-\infty}^{\infty}C^{(r,m)}_{n}e^{2\pi inx}. \end{aligned} $$

Here

14 $$ \begin{aligned}[b] C^{(r,m)}_{n}&= \int_{0}^{1}\mathit{Ch}^{(r)}_{m}\bigl( \langle x \rangle\bigr)e^{-2\pi inx}\,dx = \int_{0}^{1}\mathit{Ch}^{(r)}_{m}(x)e^{-2\pi inx}\,dx \\ &=\frac{1}{m+1} \bigl[\mathit{Ch}^{(r)}_{m+1}(x)e^{-2\pi inx} \bigr]^{1}_{0}+\frac {2\pi in}{m+1} \int_{0}^{1}\mathit{Ch}^{(r)}_{m+1}(x)e^{-2\pi inx}\,dx \\ &=\frac{1}{m+1} \bigl(\mathit{Ch}^{(r)}_{m+1}(1)-\mathit{Ch}^{(r)}_{m+1} \bigr)+\frac{2\pi in}{m+1}C^{(r,m+1)}_{n} \\ &=\frac{2}{m+1} \bigl(\mathit{Ch}^{(r-1)}_{m+1}-\mathit{Ch}^{(r)}_{m+1} \bigr)+\frac{2\pi in}{m+1}C^{(r,m+1)}_{n}. \end{aligned} $$

By virtue of replacing *m* by $m-1$ in (14), we can find

$$ \begin{aligned} \frac{2\pi in}{m}C^{(r,m)}_{n}=C^{(r,m-1)}_{n}+ \frac{2}{m} \bigl(-\mathit{Ch}^{(r-1)}_{m}+\mathit{Ch}^{(r)}_{m} \bigr). \end{aligned} $$

### Case 1

Let $n\neq0$. Then we acquire that

$$ \begin{aligned} C^{(r,m)}_{n}={}& \frac{m}{2\pi in}C^{(r,m-1)}_{n}+\frac{1}{\pi in} \bigl(\mathit{Ch}^{(r)}_{m}-\mathit{Ch}^{(r-1)}_{m} \bigr) \\ ={}&\frac{m}{2\pi in} \biggl(\frac{m-1}{2\pi in}C^{(r,m-2)}_{n}- \frac{1}{\pi in} \bigl(\mathit{Ch}^{(r)}_{m-1}-\mathit{Ch}^{(r-1)}_{m-1} \bigr) \biggr) \\ & +\frac{1}{\pi in} \bigl(\mathit{Ch}^{(r)}_{m}-\mathit{Ch}^{(r-1)}_{m} \bigr) \\ ={}&\frac{m(m-1)}{(2\pi in)^{2}}C^{(r,m-2)}_{n}+\frac{m}{2(\pi in)^{2}} \bigl(\mathit{Ch}^{(r)}_{m-1}-\mathit{Ch}^{(r-1)}_{m-1} \bigr) \\ & +\frac{1}{\pi in} \bigl(\mathit{Ch}^{(r)}_{m}-\mathit{Ch}^{(r-1)}_{m} \bigr) \\ ={}&\frac{m(m-1)}{(2\pi in)^{2}} \biggl(\frac{m-2}{2\pi in}C^{(r,m-3)}_{n}- \frac{1}{\pi in} \bigl(\mathit{Ch}^{(r)}_{m-2}-\mathit{Ch}^{(r-1)}_{m-2} \bigr) \biggr) \\ & +\frac{m}{2(\pi in)^{2}} \bigl(\mathit{Ch}^{(r)}_{m-1}-\mathit{Ch}^{(r-1)}_{m-1} \bigr)+\frac{1}{\pi in} \bigl(\mathit{Ch}^{(r)}_{m}-\mathit{Ch}^{(r-1)}_{m} \bigr) \\ ={}&\frac{m(m-1)(m-2)}{(2\pi in)^{3}}C^{(r,m-3)}_{n}+\frac{m(m-1)}{2^{2}(\pi in)^{3}} \bigl(\mathit{Ch}^{(r)}_{m-2}-\mathit{Ch}^{(r-1)}_{m-2} \bigr) \\ & +\frac{m}{2(\pi in)^{2}} \bigl(\mathit{Ch}^{(r)}_{m-1}-\mathit{Ch}^{(r-1)}_{m-1} \bigr)+\frac{1}{\pi in} \bigl(\mathit{Ch}^{(r)}_{m}-\mathit{Ch}^{(r-1)}_{m} \bigr) \\ ={}&\cdots \\ ={}&\frac{m!}{(2\pi in)^{m-1}}C^{(r,1)}_{n}+\sum _{k=1}^{m-1}\frac {2(m)_{k}}{(2\pi in)^{k}} \bigl(\mathit{Ch}^{(r)}_{m-k+1}-\mathit{Ch}^{(r-1)}_{m-k+1} \bigr). \end{aligned} $$

In addition, we observe that

$$ \begin{aligned} C^{(r,1)}_{n}&= \int_{0}^{1}\mathit{Ch}^{(r)}_{1}(x)e^{-2\pi inx}\,dx= \int _{0}^{1}\bigl(x+\mathit{Ch}^{(r)}_{1} \bigr)e^{-2\pi inx}\,dx \\ &= \int_{0}^{1}xe^{-2\pi inx}\,dx+\mathit{Ch}^{(r)}_{1} \int_{0}^{1}e^{-2\pi inx}\,dx \\ &=-\frac{1}{2\pi in} \bigl[xe^{-2\pi inx} \bigr]^{1}_{0}+ \frac{1}{2\pi in} \int_{0}^{1}e^{-2\pi inx}\,dx \\ &=-\frac{1}{2\pi in}. \end{aligned} $$

Therefore, we can derive the following equation:

$$ \begin{aligned} C^{(r,m)}_{n}&=\frac{-m!}{(2\pi in)^{m}}+ \sum_{k=1}^{m-1}\frac {2(m)_{k-1}}{(2\pi in)^{k}} \bigl(\mathit{Ch}^{(r)}_{m-k+1}-\mathit{Ch}^{(r-1)}_{m-k+1} \bigr) \\ &=\sum_{k=1}^{m}\frac{2(m)_{k-1}}{(2\pi in)^{k}} \bigl(\mathit{Ch}^{(r)}_{m-k+1}-\mathit{Ch}^{(r-1)}_{m-k+1} \bigr). \end{aligned} $$

Here, we used the fact that

$$ \mathit{Ch}^{(r)}_{1}-\mathit{Ch}^{(r-1)}_{1}=rCh_{1}-(r-1)\mathit{Ch}_{1} =\mathit{Ch}_{1}=-\frac{1}{2}. $$

Indeed,

$$ \begin{aligned} \sum_{n=0}^{\infty}\mathit{Ch}^{(r)}_{n} \frac{t^{n}}{n!}&= \biggl(\frac{2}{2+t} \biggr)\times\cdots\times \biggl( \frac{2}{2+t} \biggr) \\ &=\sum_{n=0}^{\infty} \biggl(\sum _{l_{1}+\cdots+l_{r}=n}\binom {n}{l_{1},l_{2},\ldots,l_{r}}\mathit{Ch}_{l_{1}}\mathit{Ch}_{l_{2}}\cdots \mathit{Ch}_{l_{r}} \biggr)\frac{t^{n}}{n!}. \end{aligned} $$

Accordingly, it follows that

$$ \begin{aligned} \mathit{Ch}^{(r)}_{1}&=\sum _{l_{1}+\cdots+l_{r}=1}\binom{1}{l_{1},l_{2},\ldots ,l_{r}}\mathit{Ch}_{l_{1}}\mathit{Ch}_{l_{2}} \cdots \mathit{Ch}_{l_{r}} \\ &=\mathit{Ch}_{1}+\mathit{Ch}_{1}+\cdots+\mathit{Ch}_{1}=rCh_{1}. \end{aligned} $$

### Case 2

Let $n=0$. Then we have

$$ \begin{aligned} C^{(r,m)}_{0}&= \int_{0}^{1}\mathit{Ch}^{(r)}_{m}(x)\,dx \\ &=\frac{1}{m+1} \bigl[\mathit{Ch}^{(r)}_{m+1}(1)-\mathit{Ch}^{(r)}_{m+1} \bigr]^{1}_{0} \\ &=\frac{2}{m+1} \bigl(\mathit{Ch}^{(r-1)}_{m+1}-\mathit{Ch}^{(r)}_{m+1} \bigr). \end{aligned} $$

Assume first that $\mathit{Ch}^{(r)}_{m}(1)=\mathit{Ch}^{(r)}_{m}$. Then we have $\mathit{Ch}^{(r)}_{m}(1)=\mathit{Ch}^{(r)}_{m}$ for $m\geq2$. $\mathit{Ch}^{(r)}_{m}(\langle x \rangle)$ is piecewise $C^{\infty}$ and continuous. Hence the Fourier series of $\mathit{Ch}^{(r)}_{m}(\langle x\rangle)$ converges uniformly to $\mathit{Ch}^{(r)}_{m}(\langle x\rangle)$, and

$$ \begin{aligned} \mathit{Ch}^{(r)}_{m}\bigl(\langle x \rangle\bigr)={}&\frac{2}{m+1} \bigl(\mathit{Ch}^{(r-1)}_{m+1}-\mathit{Ch}^{(r)}_{m+1} \bigr) \\ & +\sum_{\substack{n=-\infty\\ n\neq0}} \Biggl[\sum _{k=1}^{m}\frac {2(m)_{k-1}}{(2\pi in)^{k}} \bigl(\mathit{Ch}^{(r)}_{m-k+1}-\mathit{Ch}^{(r-1)}_{m-k+1} \bigr) \Biggr]e^{2\pi inx}. \end{aligned} $$

Consequently, it follows that

$$ \begin{aligned} \mathit{Ch}^{(r)}_{m}\bigl( \langle x\rangle\bigr)={}&\frac{2}{m+1} \bigl(\mathit{Ch}^{(r-1)}_{m+1}-\mathit{Ch}^{(r)}_{m+1} \bigr) \\ & +\sum_{k=1}^{m}\frac{2(m)_{k-1}}{k!} \bigl(\mathit{Ch}^{(r-1)}_{m-k+1}-\mathit{Ch}^{(r)}_{m-k+1} \bigr)\sum _{\substack{n=-\infty\\ n\neq0}}(-k!)\frac{e^{2\pi inx}}{(2\pi in)^{k}} \\ ={}&\frac{2}{m+1} \bigl(\mathit{Ch}^{(r-1)}_{m+1}-\mathit{Ch}^{(r)}_{m+1} \bigr) \\ & +\sum_{k=2}^{m}\frac{2(m)_{k-1}}{k!} \bigl(\mathit{Ch}^{(r-1)}_{m-k+1}-\mathit{Ch}^{(r)}_{m-k+1} \bigr)B_{k}\bigl(\langle x\rangle\bigr) \\ & +2 \bigl(\mathit{Ch}^{(r-1)}_{m}-\mathit{Ch}^{(r)}_{m} \bigr)\times \textstyle\begin{cases} B_{1}(\langle x\rangle)& \text{for $x\notin\mathbb{Z}$},\\ 0 & \text{for $x\in\mathbb{Z}$}. \end{cases}\displaystyle \end{aligned} $$

Thus the proof of Theorem 3 is complete.

Finally, assume that $\mathit{Ch}^{(r)}_{m}\neq \mathit{Ch}^{(r-1)}_{m}$. Then we have $\mathit{Ch}^{(r)}_{m}(1)\neq \mathit{Ch}^{(r)}_{m}$ and hence $\mathit{Ch}^{(r)}_{m}(\langle x\rangle)$ is piecewise $C^{\infty}$ and discontinuous with jump discontinuities at integers. Thus the Fourier series of $\mathit{Ch}^{(r)}_{m}(\langle x\rangle)$ converges pointwise to $\mathit{Ch}^{(r)}_{m}(\langle x\rangle)$ for $x\notin\mathbb{Z}$, and converges to $\frac{1}{2}(\mathit{Ch}^{(r)}_{m}+\mathit{Ch}^{(r)}_{m}(1))=\mathit{Ch}^{(r-1)}_{m}$ for $x\in \mathbb{Z}$. From the above considerations, the proof of Theorem 4 is complete.

## Conclusions

In this paper, the author considered the Fourier series expansion of the higher-order Daehee functions $D^{(r)}_{n}(\langle x\rangle)$ and the higher-order Changhee functions $\mathit{Ch}^{(r)}_{n}(\langle x\rangle)$ which are obtained by extending by periodicity of period 1 the higher-order Daehee polynomials $D^{(r)}_{n}(x)$ and the higher-order Changhee polynomials $\mathit{Ch}^{(r)}_{n}(x)$ on $[0,1)$, respectively. The Fourier series are explicitly determined. Depending on whether $D^{(r)}_{n}(\langle x\rangle)$ and $\mathit{Ch}^{(r)}_{n}(\langle x\rangle)$ are zero or not, the Fourier series of these functions converge uniformly or converge pointwise. In addition, the Fourier series of the higher-order Daehee functions $D^{(r)}_{n}(\langle x\rangle)$ and the higher-order Changhee functions $\mathit{Ch}^{(r)}_{n}(\langle x\rangle)$ are expressed in terms of the Bernoulli functions $B_{k}(\langle x\rangle)$. Thus we established the relations between these functions and Bernoulli functions.
